# Heroic music stimulates empowering thoughts during mind-wandering

**DOI:** 10.1038/s41598-019-46266-w

**Published:** 2019-07-16

**Authors:** Stefan Koelsch, Tobias Bashevkin, Joakim Kristensen, Jonas Tvedt, Sebastian Jentschke

**Affiliations:** University of Bergen, Department of Biological and Medical Psychology, Postboks 7807, 5020 Bergen, Norway

**Keywords:** Human behaviour, Physiology

## Abstract

It is generally well-known, and scientifically well established, that music affects emotions and moods. However, only little is known about the influence of music on thoughts. This scarcity is particularly surprising given the importance of the valence of thoughts for psychological health and well-being. We presented excerpts of heroic- and sad-sounding music to *n* = 62 individuals, and collected thought probes after each excerpt, assessing the valence and the nature of thoughts stimulated by the music. Our results show that mind-wandering emerged during listening to either type of music (heroic, sad), and that the type of music strongly influenced the thought contents during mind-wandering. Heroic-sounding music evoked more positive, exciting, constructive, and motivating thoughts, while sad-sounding music evoked more calm or demotivating thoughts. The results thus indicate that music has a strong effect on the valence of thought contents during mind-wandering, with heroic music evoking more empowering and motivating thoughts, and sad music more relaxing or depressive thoughts. These findings have important implications for the use of music in everyday life to promote health and well-being in both clinical populations and healthy individuals.

## Introduction

During the last years, psychological science has devoted increasing research efforts on differentiating two thought modes: consciously controlled thought (i.e., deliberate thought, with awareness of the control over thoughts) and spontaneous, non-intentional thoughts without awareness of the control over thoughts (often referred to as “mind-wandering”). For example, Smallwood & Schooler^1^ proposed that mind-wandering is characterized by a switch in attention from a current task to unrelated thoughts and feelings, and the term “mind-wandering” is, accordingly, often used synonymously with “task-unrelated imagery and thoughts”, “stimulus-independent thought”, “task-unrelated thought”, or “zoning out”^1–3^. Typically, the switch of attention from a current task to task-unrelated thoughts happens unintentionally and without meta-awareness, i.e. without awareness of the control over thoughts (although cases of *intentional* mind-wandering have also been proposed in the literature)^4–6^.

Many people spend a substantial amount of their time mind-wandering: For example, using an experience sampling method, Killingsworth & Gilbert reported that mind-wandering occurred in almost half of the samples^7^. Consistent with this observation, functional neuroimaging experiments investigating the “default mode network” make use of the phenomenon that, when participants are not presented with a specific task (i.e., when they are in a so-called “resting state”), individuals engage in mind-wandering^3,4^. Even when focusing on a specific task, many people often get distracted by mind-wandering^1–4,7^. The disruptive effects of mind-wandering on primary tasks have been suggested to be mainly due to lack of meta-awareness (i.e., lack of awareness of the control of thought) and perceptual decoupling (i.e., disengaging attention from perception)^4,8–11^.

It has been debated whether the thought content of mind-wandering has a positive or a negative bias (e.g., Killingsworth & Gilbert reported a negative bias, whereas Andrews-Hanna *et al*. reported a positive bias in a recent meta-analysis)^7,12^. However, there is strong consensus that mind-wandering can have positive or negative thought content. Importantly, mind-wandering with negative thought content is often associated with negative moods: mind-wandering with negative thought content can cause negative mood^7,13^, and negative mood can cause mind-wandering with negative thought content^14,15^. This aspect makes mind-wandering also clinically relevant, especially with regard to rumination (a typical symptom of depression) or pathological anxiety^16,17^. Mind-wandering with positive thought content, on the other hand, can have positive effects such as facilitation of creative ideas and problem solving^18^, and lead to mood improvements^19^.

Given that many individuals spend a substantial amount of their time mind-wandering, and that the thought contents during mind-wandering can lead to positive or negative influences on the mood of individuals, it is of interest and relevant to understand the factors that influence the valence of thoughts during mind-wandering. A recent study from Taruffi *et al*. reported that sad-sounding music evoked more mind-wandering than happy-sounding music, and that happy-sounding music led to more positive thoughts during mind-wandering than sad music^20^. That study used a probe-caught thought sampling method, in which individuals were presented with either sad- or happy-sounding music, and intermittently asked about their current mental state. Consistent with the observation that sad-sounding music evoked more mind-wandering than happy-sounding music, a functional neuroimaging experiment using the same stimuli suggested that the midline-core of the default mode network (including orbitofrontal, anterior cingulate, and posterior cingulate cortex) showed increased functional centrality (as measured using eigenvector centrality mapping) during sad-sounding than during happy-sounding music^20^.

Motivated by the results of that study^20^, we investigated in the present study effects of “heroic”, or “empowering” music, in comparison with sad-sounding music, on mind-wandering. In particular, we aimed at testing if these different types of music affect the valence of the thought contents during mind-wandering. Already Plato and Aristotle wrote about music’s capacity to evoke braveness, self-confidence, and enthusiasm^21^. However, despite its age-long relevance, this capacity of music has received little scientific attention. Hsu *et al*. reported that empowering music’s capabilities to create subjective feelings of power implicitly activated power-related thoughts and behaviors (e.g., implicitly activating the construct of power, abstract thinking, illusory control, and the tendency to “move first”)^22^. Such potentially beneficial effects of empowering music have relevance for both healthy individuals and clinical populations. In healthy individuals, empowering music has been reported to promote self-confidence, possibly by triggering positive thoughts and appraisals related to aspects of self-confidence^23^. Moreover, in everyday life, empowering music can potentially help motivating and encouraging to engage in tasks, and to reduce distraction by negative thoughts when concentrating on tasks. In populations with psychopathology (e.g., in patients with depression), empowering music might have the capacity to help patients with feelings of reduced energy and helplessness.

According to Zentner *et al*.^24^, empowering music is characterized by vitality, and can have different facets such as sounding energetic, triumphant, fiery, strong and heroic. In the present study, we opted to focus on effects of music that sounds heroic, and by association energetic and strong (rather than triumphant and fiery). This choice afforded us to match the tempo of our heroic music stimuli with the tempo of sad-sounding stimuli. This seemed particularly relevant because the study by Taruffi *et al*.^20^, which used happy- and sad-sounding music, faced the difficulty that these two types of emotional expression in music are characterized by different tempi. Although effects of faster music on mind-wandering frequency were compared with those of slower music, effects of tempo on the contents of thoughts were not analyzed further. Thus, it is an open question if the emotional expression of music (or perhaps simply the tempo, or amount, of incoming perceptual information) influences thought contents.

In the present study, *n* = 62 participants were presented, in six different trials, with 2-min. excerpts of heroic- or sad-sounding music (matched in tempo, loudness, and orchestration). After each excerpt, they answered an 11-item questionnaire designed to assess the extent of mind-wandering, and characterize the contents of thoughts, for example with regard to valence, arousal, constructiveness, and motivation (for details see Methods and Supplementary Table S5). In addition, participants also had the opportunity to write a brief description of their thoughts. At the beginning of the experiment, and after each thought probe, the affect of the participants was assessed using a 10-item questionnaire^25^. Throughout the experiment, the heart rate of participants was measured to control for physiological arousal effects of the music stimuli. We hypothesized that heroic music, compared with sad music, would evoke more positive mood, and lead to lower mind-wandering frequency, as well as a more positive valence of the contents of thoughts. In addition, we aimed at testing whether heroic music would elicit more constructive thoughts during mind-wandering, and motivate to behave more actively.

## Results

To restrict our analysis to trials in which “mind-wandering” was elicited, we selected trials in which responses to the questionnaire item “How much were you thinking about the music vs. something else?” were −1 or higher (on a scale of −3 to 3; for analyses with different cutoff-values see Supplementary Text S1). Participants reported mind-wandering after 73.1% of all trials, with a very similar prevalence of mind-wandering after listening to heroic (72.6%) and sad excerpts (73.7%). An ANOVA for repeated measures, using the within-subject factors emotional expression (heroic vs. sad music) and tempo (slow, medium or fast) as well as the between-subjects factor presentation order (heroic first vs. sad first), revealed no significant difference in the degree of mind-wandering between pieces with different emotional expression (*p* = 0.54) or with different tempi (*p* = 0.27).

**Figure 1:**
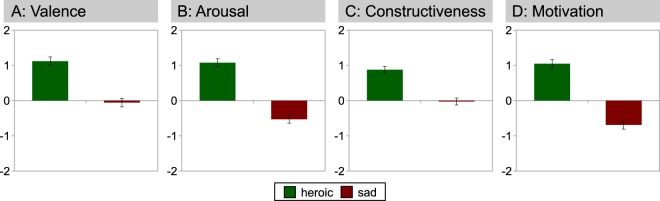
Mean differences in thought content ratings reported after listening to heroic vs. sad musical excerpts (scale range was −3 to +3, error bars indicate SEM).

To investigate the thought contents of mind-wandering, we then selected those trials that elicited mind wandering (using the cutoff value of −1 reported above; additional analyses with different cutoff values, as well as analyses including all trials are reported in Supplementary Text S1 and Table S2). During listening to heroic music, thought contents were more positive than during sad music (during which the valence of thought contents was on average neutral, Fig. 1A). Moreover, thought contents were more exciting (or about something that would make participants more excited) during heroic music, and more calm (or about something that would make participants calm) during sad music (Fig. 1B). Thought contents were also more constructive during heroic than sad music (Fig. 1C), and more motivating during heroic, but more demotivating during sad music (see Fig. 1D; for exact wording of questionnaire items see items #6, #7, #10 & #11 in Supplementary Table S5). Meta-awareness and awareness of control over thoughts (both included in our definition of mind-wandering) were similar in both conditions: Mean ratings for controlled focus of attention (assessed with questionnaire item #2) were −0.27 (*SD* = 1.65) during heroic, and −0.29 (*SD* = 1.73) during sad music. Mean ratings for meta-awareness (assessed with questionnaire item #3) were −0.3 (*SD* = 1.75) during heroic, and −0.28 (*SD* = 1.68) during sad music. ANOVAs testing for the influence of the within-subject factors emotional expression and tempo of the musical excerpts on the thought-content items revealed significant effects on valence, arousal, constructiveness, and motivation (cf. Table 1A), each with large effect sizes for the factor emotional expression. It is worth noting that the item used for assessing mind-wandering (“How much were you thinking about the music vs. something else?”) did neither correlate with meta-awareness, nor with controlled focus of attention, but only with the item “Were your thoughts related to something that is personally relevant?” (*ρ* = 0.25, *p* < 0.001).

**Table 1:** Overview of the results from analyses of variance for items assessing thought content and positive-negative affect, including the trials where participants reported mind-wandering.

Item topic	Significant effects	F-Value	Sign.	Effect size
**A: Items assessing thought-content (while controlling for the tempo of the music excerpts)**				
«Valence»	heroic-sad	*F*_(1,55.86)_ = 34.74	*p* < 0.0001	*d* = 1.58
«Arousal»	heroic-sad	*F*_(1,56.31)_ = 72.89	*p* < 0.0001	*d* = 2.27
«Motivation»	heroic-sad	*F*_(1,54.12)_ = 49.84	*p* < 0.0001	*d* = 1.92
«Constructiveness»	heroic-sad	*F*_(1,54.42)_ = 12.53	*p* = 0.0008	*d* = 0.96
«Meta-awareness»	no sign. effects			
«Controlled focus of attention»	no sign. effects			
«Self-involvement»	no sign. effects			
«Involvement of others»	no sign. effects			
«Temporal orientation of thoughts»	no sign. effects			
«Relevance to current life-situation»	no sign. effects			
**B: Items assessing thought-content (while controlling for physiological arousal)**				
«Valence»	heroic-sad	*F*_(1,69.31)_ = 38.01	*p* < 0.0001	*d* = 1.50
«Arousal»	heroic-sad	*F*_(1,65.02)_ = 73.35	*p* < 0.0001	*d* = 2.15
«Motivation»	heroic-sad	*F*_(1,60.64)_ = 48.97	*p* < 0.0001	*d* = 1.81
«Constructiveness»	heroic-sad	*F*_(1,60.74)_ = 17.71	*p* < 0.0001	*d* = 1.09
«Meta-awareness»	no sign. effects			
«Controlled focus of attention»	no sign. effects			
«Self-involvement»	no sign. effects			
«Involvement of others»	no sign. effects			
«Temporal orientation of thoughts»	no sign. effects			
«Relevance to current life-situation»	no sign. effects			
**C: Positive-negative affect scale (while controlling for physiological arousal)** ^25^				
«positive affect»	heroic-sad	*F*_(1,58.06)_ = 29.11	*p* < 0.0001	*d* = 1.42
«negative affect»	heroic-sad	*F*_(1,61.00)_ = 9.73	*p* = 0.0028	*d* = 0.80
«inspired»	heroic-sad	*F*_(1,62.59)_ = 33.95	*p* < 0.0001	*d* = 1.47
«determined»	heroic-sad	*F*_(1,60.18)_ = 31.62	*p* < 0.0001	*d* = 1.45
«active»	heroic-sad	*F*_(1,59.65)_ = 29.02	*p* < 0.0001	*d* = 1.39
«afraid»	heroic-sad	*F*_(1,59.44)_ = 14.46	*p* = 0.0003	*d* = 0.99
«alert»	heroic-sad	*F*_(1,58.95)_ = 11.41	*p* = 0.0013	*d* = 0.88
«upset»	no sign. effects			
«hostile»	no sign. effects			
«ashamed»	no sign. effects			
«nervous»	no sign. effects			
«attentive»	no sign. effects			

Follow-up analyses explored whether ratings of the items that showed a significant effect of heroic-sad (valence, arousal, constructiveness and motivation) differed from neutral in the two conditions (heroic, sad). The Bonferroni-corrected significance level was 0.05/8 = 0.0063 (four significant effects × two conditions = 8). For the heroic condition, valence (*t*_(61)_ = 10.83; *p* < 0.0001), arousal (*t*_(61)_ = 8.66; *p* < 0.0001), constructiveness (*t*_(61)_ = 8.73; *p* < 0.0001), and motivation (*t*_(61)_ = 8.76; *p* < 0.0001) were all significantly different from neutral. For the sad condition, arousal (*t*_(61)_ = −5.10; *p* < 0.0001) and motivation (*t*_(61)_ = −6.48; *p* < 0.0001) were significantly different from neutral; valence and constructiveness were not.

To determine whether the arousal difference of thought contents (as reported above) was associated with peripheral-physiological changes, the mean heart rate was evaluated as an indicator of physiological arousal. When listening to heroic music, heart rate was higher than during sad music, which is in accordance with the higher arousal ratings. An ANOVA for repeated measurements on the mean heart rate with the within-subject factors emotional expression (heroic vs. sad) and tempo (slow, medium, and fast), and the presentation order (heroic first vs. sad first) as between-subjects factor revealed a significant effect (with large effect size) of emotional expression (*F*_(1,57)_ = 16.68, *p* = 0.0001, *d* = 1.08), and a significant interaction between emotional expression and presentation order (*F*_(1,57)_ = 7.98, *p* = 0.0065, *d* = 0.75): Arousal did not differ at baseline between the two presentation orders (heroic first vs. sad first; i.e. before the presentation of the first music excerpt). However, physiological arousal was generally slightly higher when heroic pieces were presented first, and there was a larger difference in heart rate between heroic and sad pieces in the group that got presented with heroic pieces first.

To control for the influence of physiological arousal on thought-content, the ANOVAs reported above (cf. Table 1A) were repeated with physiological arousal as covariate. Because the tempo of the musical excerpts strongly covaried with physiological arousal, the factor tempo was replaced with the factor physiological arousal (i.e., the ANOVAs reported below had the factor emotional expression, and physiological arousal as covariate; recall that the average tempo of heroic excerpts did not differ from the tempo of sad excerpts). Generally, the results of the ANOVAs controlling for physiological arousal are consistent with the previous ANOVAs (controlling for tempo), confirming the significant effects for emotional expression on thought content with regard to valence, arousal, constructiveness, and motivation (see Table 1B).

**Figure 2:**
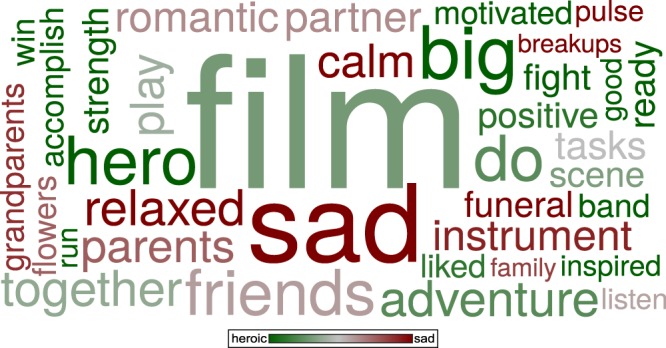
World-cloud using the most common words used in the free responses after listening to heroic- and sad-sounding music. The font size reflects the frequency of occurrence, the color indicates whether the word was more often used after listening to heroic musical excerpts (dark green) or after sad musical excerpts (dark red).

Participants also described their thought contents in a free response format. From the different words reported in these descriptions (including all trials in which any response was provided by a participant), a word-cloud was created (Fig. 2; for details see *Methods*). The word-cloud revealed that heroic music elicited thoughts that were more related to activities and motivation (such as “hero”, “accomplish”, and “strength”), whereas sad music elicited thoughts more related to sadness or relaxation (such as “sad”, “relaxed”, and “calm”). Using the *Affective Norms for English Words*^26^, values for valence and arousal for words in the free word response were identified, and compared between the heroic and sad condition using an ANOVA. This analysis indicated differences in both valence (*F*_(1,229.92)_ = 57.67, *p* < 0.0001, *d* = 0.96) and arousal (*F*_(1,84.20)_ = 92.56, *p* < 0.0001, *d* = 1.21), with higher valence (i.e. more positive) and higher arousal values for words describing thoughts during heroic compared with sad music.

To further specify the nature of thoughts during music listening, we also inspected the free responses with regard to thoughts that included problem solving (e.g. “how I could help my friend to tidy her home”), planning (e.g. “whether I will eat at home or at the student center”), or reasoning involving logical connectives such as “if-then”, “either-or”, “only if” etc. Out of 372 free responses, we found only eight such responses (four in each condition).

**Figure 3:**
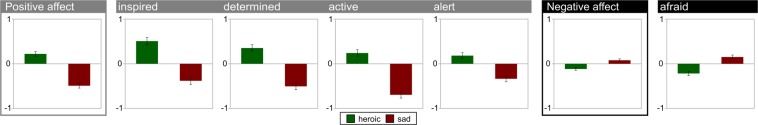
Participants’ mean responses (relative to their baseline values) on the Positive and Negative Affect Schedule (PANAS) after listening to heroic vs. sad musical excerpts (error bars indicate SEM). Enclosed boxes indicate subscale values, the other panels indicate item values.

To assess effects of the music on the mood of participants, we obtained affect ratings at the beginning of the experiment and after each music excerpt using the Positive and Negative Affect Schedule (PANAS)^25^. In the following, we again report results for trials in which mind-wandering occured (i.e., trials with a cutoff of −1 on the questionnaire item “How much were you thinking about the music vs. something else?”); an analysis including all trials indicated essentially the same results (cf. Supplementary Table S2). Figure 3 shows that positive mood (as measured with the subscale “positive affect”) was significantly higher after listening to heroic as compared with sad music. Vice versa, negative mood (as measured with the subscale “negative affect”) was significantly higher after sad as compared with heroic music. The same results were obtained for the majority of the items assessing affect quality (see Table 1C for details). Separate ANOVAs with the factors emotional expression while controlling for physiological arousal (using the mean heart rate as covariate) were conducted for each subscale as well as for every item (resulting in a Bonferroni-corrected significance level of *p* = 0.0042). Significant differences between heroic and sad music were obtained for both positive and negative affect subscales. On the item level, significant effects of emotional expression were found for the positive affect items: «inspired», «determined», «active» and «alert», as well as for the negative affect item: «afraid».

Follow-up analyses explored whether these subscale and item values also differed significantly from the baseline ratings (Bonferroni-corrected significance level was 0.0035). For the heroic condition, positive affect (*t*_(61)_ = 3.15; *p* = 0.0025), negative affect (*t*_(61)_ = −3.20; *p* = 0.0022), feeling inspired (*t*_(61)_ = 3.77; *p* = 0.0004), and feeling afraid (*t*_(61)_ = −3.36; *p* = 0.0013) differed significantly from the baseline assessment (no difference from baseline values was observed for feeling alert, active, and determined). For the sad condition, positive affect (*t*_(61)_ = −5.35; *p* < 0.0001), feeling determined (*t*_(61)_ = −3.40; *p* = 0.0012), and feeling active (*t*_(61)_ = −4.91; *p* < 0.0001) differed significantly from the baseline assessment (no difference from baseline values was observed for negative affect, nor for feeling alert, inspired, or afraid).

## Discussion

As expected, the two types of music (heroic and sad) had large, and highly significant, effects on the emotional state (as reflected in the PANAS ratings). This does not come as a surprise, given the well-established literature on the induction of emotions and moods with music^24,27–29^. In specific, heroic-sounding music influenced the emotional state such that participants felt more positive, more inspired, active, alert, and less afraid, compared with listening to sad-sounding music. Even in comparison to the baseline values, heroic music elicited more positive (and less negative) affect, made participants feel more inspired, and less afraid. This suggests that heroic-sounding music can be used in everyday life to feel more positive, motivated, and courageous. Sad music, on the other hand, reduced positive mood, and diminished feeling determined and active.

Importantly, beyond the well-established effects of music on emotions and moods, our study shows that, while both types of music (heroic, sad) evoked mind-wandering to a similar degree, the quality of the thoughts during mind-wandering was very different between the two types of music. Our results show large (and highly significant) effects of heroic- and sad-sounding music on the contents of thoughts during music listening: During listening to heroic music (compared with sad music), the valence of thoughts was more positive, the thoughts were more activating, and they were more constructive as well as more motivating. During sad music, the thought contents had on average a neutral valence, had the potential to calm (i.e., a negative arousal), and were rather demotivating (i.e., motivation ratings were lower than neutral values).

Thus, both subjective feeling (as indicated in the PANAS ratings) and thought contents of participants were more empowering after heroic music. Importantly, the analysis of the free responses shows that ratings of thought contents were not simply biased by the emotion ratings (such that, e.g., in a positive emotional state individuals would tend to provide more positive responses in general, i.e., even on scales unrelated to emotions such as scales assessing cognitive evaluations). Instead, the free responses demonstrate that the actual thoughts (not merely the ratings on the scales) were more positive and more motivating. For example, during heroic music, participants’ thoughts and verbal responses were more about something motivating, heroic, strong, and about accomplishing something. Thus participants’ thoughts (not merely their emotions) were actually more empowering. On the other hand, thoughts during sad music were more about something relaxing, but also more often about something demotivating or sad (such as a breakup or a funeral).

These observations are notable because the main interest of affective science in cognitive processes has so far focused on appraisal processes, i.e. on evaluative cognitive processes as *antecedents* of emotions (such processes evaluate, e.g., if a stimulus is a potential reward or penalty)^30^. By contrast, our study investigates thoughts (in response to a stimulus) *beyond* appraisal processes, and finds that the contents of these thoughts can be influenced systematically by music. Thus, in addition to the well-established influence of thoughts on emotions, our data add to the rather scant empirical literature showing that emotions impact on thoughts^31^.

Our results are consistent with the results of the study by Taruffi *et al*.^20^, which showed more positive thoughts during happy-sounding music compared with sad-sounding music. In that study, however, it could not be ruled out that effects of happy and sad music were at least in part due to tempo differences, because happy music was on average faster than sad music. In our study, effects could not be due to the tempo of the music because the average tempo of heroic and sad pieces was identical. Therefore, the combined evidence of the findings by Taruffi *et al*. and the results of the present study indicate that music has a clear and large effect on the valence of thought contents.

The heart rate of participants was higher during listening to heroic (compared with sad) music. This is well in accordance with the arousing effects of activating music, and calming effects of sad and peaceful music^32^. Importantly, even when controlling for the influence of physiological arousal on thought-content (by using heart rate as an indicator of arousal as covariate in the analysis of thought contents) the effects of music on thought contents remained, with large effect sizes.

The conclusion that music influences thought contents is consistent with studies showing effects of music on cognitive-evaluative processes, such as effects of music on brightness judgements (individuals perceive a grey square as brighter after listening to happy-, as compared with sad-sounding music)^33^, or less risk aversion bias during decision making after listening to happy (compared with sad) music^34^. These studies, however, did not report any effects of music on the contents of thoughts, nor their valence. Nevertheless, other previous studies have reported that sad music can evoke mind-wandering^20^, and rumination (i.e. recurring thoughts with negative valence) in individuals with depression or a tendency for depression^35–38^. Due to such effects, it appears that listening to sad music is a risk factor in individuals prone to depression, and that listening to sad music may even trigger a relapse in individuals with depression. Heroic music, on the other hand, might be used to help individuals with depression or with tendencies to depression. Because individuals with depression usually show strong preferences for sad music^35,38^, it seems advisable for such individuals to listen to sad music only within a sequence of songs leading to songs with positive emotional expression, such as heroic and empowering songs.

Beyond patients with depression, or individuals prone to depression, the potential of heroic-sounding music to empower thoughts has also potential for the everyday use of music in healthy individuals, for example by using empowering music to improve constructive thought, and set plans into action (due to thoughts being more motivating and activating). Moreover, a more passive than active behavior has been taken as a behavioral effect of low self-confidence (and an active strategy of fostering self-confidence has been reported to be beneficial, especially in the face of very difficult tasks and if one’s willpower is not that strong)^39^. Therefore, listening to heroic-sounding music appears to be an effective strategy to heighten low self-confidence as well as improve constructive thought, and thus to contribute to psychological health, human flourishing and resilience^40^.

The exact mechanisms through which music influences thoughts are not known. One possibility is that the emotional expression of the heroic music (characterized by emotional expression of power, inspiration, heroism, and courage) led to both emotional resonance (or “emotional contagion”) as well as “cognitive resonance” (or “cognitive contagion”). Such cognitive resonance has been discussed in the music-psychological literature under the term “visual imagery”, i.e., elicitation of mental images (such as scenes of nature, individuals celebrating, etc.) during music listening^27^. However, only little empirical evidence is available on this topic. It is also possible that thoughts are mediated by emotions elicited by music (or vice versa). These issues remain to be specified.

## Limitations and Future Directions

The focus of our study was the influence of music on non-intentional, spontaneous thought. This thought mode is often referred to as “mind-wandering”, but a definition of mind-wandering is challenging: there is no consensus about a definition of mind-wandering, and some definitions are mutually exclusive^5,6^. We suggest here to differentiate mind-wandering from deliberate, effortful thinking, such as deliberate problem solving, planning, or logical thinking. The differentiation of these two thought modes is reminiscent of Kahneman’s concept of “System 1” (operating without intention, and generating fast and effortless thoughts) and “System 2” (set in motion by intention, and generating thoughts in a seemingly slow and arduous manner^41^). Critically, only “System 2” is capable of performing logical operations. Thus, “System 2” generates the type of thinking that has been investigated traditionally in cognitive science under the labels of “problem solving”, “planning”, or “reasoning”. “System 1” thoughts have been investigated mainly with a focus on the bias of judgements. We believe, however, that “mind-wandering” often, if not typically, taps into a thought mode that can be characterized by “System 1” qualities. Therefore, a conception of mind-wandering as “System 1” thought mode could help a clearer definition of “mind-wandering”, and thus help to demarcate mind-wandering from “System 2” thinking.

However, assessing spontaneous, non-intentional thought is challenging: In our study, two of the items supposed to assess mind-wandering, namely “Did you feel like you had control over where your thoughts went?” and “How aware were you of where your attention was focused?”, were apparently not very well understood by our participants: The mean ratings of these items were not different from neutral, they had only relatively little variation, and they did not correlate significantly with the item “How much were you thinking about the music vs. something else?” It thus appears that two of our three items supposed to capture mind-wandering were not sophisticated enough to capture mind-wandering, and to disentangle it from other cognitive processes. Nevertheless, our analysis of the free responses suggests that only about 2% of the reported thoughts involved logical reasoning. Thus, while participants reported thoughts in the majority of trials, participants apparently engaged only rarely in consciously controlled (“System 2”) thinking.

Finally, we note that music has a strong potential to stimulate visual imagery (both intentional and unintentional); such visual images are also thoughts (in our study, e.g., “I was thinking about being at a summer cabin by the ocean” or “I was thinking about climbing and reaching the summit of mountains”). Thus, “mind-wandering”, as we understand it here, can include, or comprise of, visual imagery. Because any type of music can probably elicit visual imagery, the extent of imagery did likely not differ between sad and heroic music in our study (while the valence and the arousal of such images differed between conditions). This might be one reason why we did not find a difference in mind-wandering frequency between heroic and sad music, in contrast to our previous study^20^. Thus, we did not replicate that particular finding of our previous study.

## Conclusions

In conclusion, our results show that both heroic- and sad-sounding music have the potential to evoke mind-wandering. The findings demonstrate that music has a large effect on the valence of thought contents during mind-wandering, with heroic-sounding music evoking more positive, exciting, constructive, and motivating thoughts, and sad-sounding music evoking more calm or demotivating thoughts. These cognitive effects were observed alongside with effects on the mood of the participants: heroic music elicited more positive (and less negative) affect, made participants feel more inspired, and less afraid, while sad music reduced positive mood, and diminished feeling determined and active. Our results contribute to a new research field, namely the investigation of effects of music on thoughts, in particular on thought mode (such as mind-wandering) and thought content. Such research will have major implications for the use of music in both patients (e.g., with regard to using heroic music to support positive thoughts in individuals with depression) and healthy individuals (e.g., with regard to using heroic music to support constructive thoughts, motivation, and self-confidence).

## Methods

### Participants

Sixty-two participants (37 females, mean age = 24.9 years, SD = 9.7) took part in the experiment. None of the participants was a professional musician, 38% reported to have no musical training, 49% reported to have less than, and 13% of the participants more than five years of extra-curricular music lessons. Participants were compensated with a gift card (80 NOK, about 10 USD). Signed informed consent was obtained from all participants, and the study was carried out according to the Declaration of Helsinki. Because the experimental procedure was non-invasive and did not involve the collection of any personal information that could be used to identify the participants, no further approval was required (according to the Norwegian Centre for Research Data; NSD).

### Stimuli and material

The stimulus-set consisted of six musical excerpts (Supplementary Table S3), selected based on a pilot study (for details see below). The stimuli were organized in three pairs, each pair consisting of one excerpt with a heroic and one excerpt with a sad emotional expression. The stimuli within each pair had equal tempo to ensure that any observed effects resulted only from the different emotional expression of the music, and not the tempo. The tempo of the excerpts belonging to the stimulus pairs was “slow” (64 beats per minute; BPM) for one pair, “medium” (95 BPM) for another pair, and “fast” (115 BPM) for the third pair. The presentation order of pairs, as well as whether the heroic or the sad excerpt within the pairs was presented first, was counter-balanced across subjects. This arrangement also ensured that the participants were not exposed to excerpts with the same emotional expression in succession. Each excerpt had a duration of two minutes, with a stable emotional expression from start to end. The stimuli were adjusted to the same loudness and had a 1.5-second fade in and -out. None of the stimuli contained any lyrics, and all pieces were orchestral, neo-orchestral, or string-orchestral music. Stimuli were selected out of 15 relatively unknown musical pieces based on a pilot study with 43 participants (for details see Supplementary Text S2).

The music stimulus and questionnaires were presented using PsychoPy (version 1.85.3)^42^ on two Lenovo laptops (G500 and B560). All of the instructions and questions presented during the experiment were in Norwegian. The participants listened to the music through headphones (Sennheiser DT770 PRO or Sony MDR-1000X). They responded to the questionnaires with the numeric buttons, and the whole keyboard for answering the free response task. An electrocardiogram (ECG) was acquired from the extremity leads using a made-to-order ECG-device (Research and Transfer Center at the University of Applied Sciences Leipzig, Germany). The ECG served to assess heart rate as an objective measure of physiological arousal during the music exposure.

### Procedure

Participants were seated in a comfortable chair with pillows for neck and lumbar support. The chairs were placed inside a semi-enclosed booth, so that the participants could not see the experimenters during the experiment. A footstool was provided to support and elevate their legs. The laptop was placed on a removable table over the participant’s lap.

Participants were first informed about the experiment and gave signed informed consent, then they answered a questionnaire concerning their musical background. Before the experiment, four electrodes for ECG-measurements were fitted on the extremities (left and right upper arm and left and right shinbone). The participants were then informed that the experiment was about music and relaxation, and instructed on how to use the keyboard and the different rating scales. They were asked to relax, follow the instructions on the screen, listen to the music passively, and remain still during the music session and the resting period to reduce artifacts in the ECG-recordings. Before the first musical excerpts was presented, participants answered the 10-items International positive and negative affect schedule short-form (I-PANAS-SF^25^; translated to Norwegian, and referred to as PANAS throughout this article) to acquire a baseline of the participants’ affective state.

The experiment encompassed 6 trials with three pairs of heroic and sad pieces. Before the presentation of each musical excerpt within a trial, the participants were instructed to sit back, relax and close their eyes to promote relaxation and perceptual decoupling. After two minutes of music-listening, a thought sampling probe was obtained, for which the participants were instructed to hold on to the last thought they had before the music stopped. They were then asked to answer items of a questionnaire designed to measure: if (or to what degree) the participants were mind-wandering; controlled focus of attention; meta-awareness; self-involvement; involvement of others; valence; arousal; temporal orientation of thoughts; relevance to current life-reality; as well as the constructiveness and motivational properties of the thought-content (the wording of all items is listed in Supplementary Table S5). While the first nine items were taken from our previous study^20^, the items on constructiveness and motivation were added to assess possible empowering effects of the heroic music on the contents of thoughts. Then, the participants were asked to write a short description of what they were thinking about (“free writing task”). Each trial ended with the 10-item PANAS, to examine (1) whether the different musical stimuli were able to elicit different affective states, and (2) whether thought-contents reflected the participant’s affective state. Excerpts with either heroic or sad emotional expression were presented in alternation in each new trial. The experiment finished with a five-minute resting period to measure mean heart rate and heart rate variability^43^, before the participants were debriefed and given contact information, in case of any inquiries or concerns. The total duration of the experimental session was approximately 45 minutes.

### Data analysis

Four different classes of analyses were carried out, assessing: (a) the occurrence of mind wandering, (b) physiological arousal (assessed using the mean heart rate), (c) thought content and (d) positive vs. negative affect. Before analyzing the data, the scales for the items assessing thought content (which ranged initially from 1 to 7) were recoded from −3 to +3 to better differentiate between negative, neutral and positive responses. For the scales assessing positive vs. negative affect, the baseline assessment of each scale (acquired before listening to the musical excerpts) was subtracted from the assessments after listening to each excerpt.

All data were analyzed using analyses of variance (ANOVA) employing the General Linear Model. Whenever these comparisons involved several items, appropriate Bonferroni-correction was used. The threshold depended on the number of items: For the ten items assessing thought-content the significance threshold was set to *p* = 0.0050 (*p* = 0.05/10). For the two subscales and the ten individual items assessing positive-negative affect it was set to *p* = 0.0042 (*p* = 0.05/12; because the correction diminished the significance thresholds by a factor of around 10, we report four decimal numbers for the *p*-values). An ANOVA for repeated measurements was used to assess what influence the emotional expression, and the tempo, of the musical excerpts had on the occurrence of mind-wandering. The model used the emotional expression (heroic vs. sad) and the tempo of the music (slow, medium, and fast) as within-subject factors, and the presentation order (heroic first vs. sad first) as between-subjects factor. A repeated-measurement ANOVA with the same factors (emotional expression, tempo and presentation order) was used to explore the physiological arousal (assessed via the mean heart rate).

Analyses evaluating the thought-content and PANAS items were restricted to trials where mind-wandering occurred, responses within trials without mind-wandering were excluded. The items eliciting mind-wandering were reorganized so that each item represented one measurement point. The reorganization required to control for the participants’ mean, given that there were six measurements for each participant (two emotional expressions × three tempi). Therefore, in the ANOVAs assessing thought-content, “participant-ID” served as random factor of no interest. In the ANOVAs assessing positive vs. negative affect the subtraction of the individual baseline measure served as control for the participants’ mean.

For the items assessing thought-content, two sets of models were used in the analyses; either of them using univariate ANOVAs. The first set of models used the within-subject factors emotional expression and tempo, while the participant-ID served as random factor of no interest. A second set of models evaluated whether the thought-content was modulated by physiological arousal. Given that tempo and physiological arousal covaried strongly, it was not possible to include both factors. Therefore, the second set of models employed the within-subject factors emotional expression and physiological arousal as well as participant-ID as random factor of no interest. For the items assessing positive vs. negative affect, only one set of models controlling for physiological arousal was used. These univariate ANOVAs used the within-subject factors emotional expression and physiological arousal.

Whenever a significant main effect for items assessing thought-content or affect (as measured with the PANAS) was obtained, follow-up analyses using one-sample *t*-tests were employed. For the items assessing thought-content, these tests evaluated whether values differed from a neutral response (i.e. zero), and for PANAS-items the tests evaluated whether values differed from the baseline values. For these models, appropriate Bonferroni-correction served to control for multiple comparisons. For items assessing thought-content (with four significant main effects × two conditions = 8 comparisons) the Bonferroni-corrected significance level was set to 0.0063 (0.05/8); for items assessing positive and negative affect (with seven significant main effects × two conditions = 14 comparisons) it was set to 0.0035 (0.05/14).

Similar to our previous study^20^, a word-cloud was created depicting the most common words in the participants’ free responses^44,45^. To this end, the frequency of word occurrences in the texts of the free writing task was computed. Misspelled words were corrected, and synonyms or related terms combined into a common denominator (e.g., “father”, “dad”, “mom”, and “mother” were combined into “parents”). Afterwards, words were removed if they were contained in the presented items or the instructions (e.g., “music”, “thinking”, “I”, “me”) or non-content words (e.g., “this”, “with”). For the final list of words used to create the word cloud, only words with at least six occurrences were chosen. The list contained (a) the word, (b) its frequency of occurrence (determining the word size in Fig. 2; sum of both conditions), and (c) whether the word appeared more often in one than the other condition (occurrences after heroic music divided by the total occurrences, determining the word color in Fig. 2).

## Supplementary information

S1 Text

S2 Text

S1 Table

S2 Table

S3 Table

S4 Table

S5 Table

## References

[1] Smallwood J, Schooler JW (2015). The science of mind wandering: empirically navigating the stream of consciousness. Annu. review psychology.

[2] Schooler, J. W. Zoning out while reading: Evidence for dissociations between experience and metaconsciousness jonathan w. schooler, erik d. reichle, and david v. halpern. In Levin, D. T. (ed.) *Thinking and seeing: Visual metacognition in adults and children*, vol. 203 (2004).

[3] Andrews-Hanna JR, Smallwood J, Spreng RN (2014). The default network and self-generated thought: component processes, dynamic control, and clinical relevance. Annals New York Acad. Sci..

[4] Seli P, Risko EF, Smilek D, Schacter DL (2016). Mind-wandering with and without intention. Trends cognitive sciences.

[5] Seli P (2018). Mind-wandering as a natural kind: A family-resemblances view. Trends cognitive sciences.

[6] Christoff K (2018). Mind-wandering as a scientific concept: cutting through the definitional haze. Trends Cogn. Sci..

[7] Killingsworth MA, Gilbert DT (2010). A wandering mind is an unhappy mind. Sci..

[8] Schooler JW (2011). Meta-awareness, perceptual decoupling and the wandering mind. Trends Cogn. Sci..

[9] Smallwood J, Andrews-Hanna J (2013). Not all minds that wander are lost: the importance of a balanced perspective on the mind-wandering state. Front. psychology.

[10] Smallwood J, Beach E, Schooler JW, Handy TC (2008). Going awol in the brain: Mind wandering reduces cortical analysis of external events. J. cognitive neuroscience.

[11] Smallwood J, Schooler JW (2006). The restless mind. Psychol. bulletin.

[12] Fox, K. C. *et al*. Affective neuroscience of self-generated thought. *Annals New York Acad. Sci*. (in press). 10.1111/nyas.13740 29754412

[13] Ruby FJ, Smallwood J, Engen H, Singer T (2013). How self-generated thought shapes mood – the relation between mind-wandering and mood depends on the socio-temporal content of thoughts. PloS one.

[14] Smallwood J, Fitzgerald A, Miles LK, Phillips LH (2009). Shifting moods, wandering minds: negative moods lead the mind to wander. Emot..

[15] Smallwood J, O’Connor RC (2011). Imprisoned by the past: unhappy moods lead to a retrospective bias to mind wandering. Cogn. & emotion.

[16] Hoffmann F, Banzhaf C, Kanske P, Bermpohl F, Singer T (2016). Where the depressed mind wanders: Self-generated thought patterns as assessed through experience sampling as a state marker of depression. J. affective disorders.

[17] Ottaviani C, Shapiro D, Couyoumdjian A (2013). Flexibility as the key for somatic health: From mind wandering to perseverative cognition. Biol. psychology.

[18] Baird, B. *et al*. Inspired by distraction mind wandering facilitates creative incubation. *Psychol. Sci*. 0956797612446024 (2012). 10.1177/0956797612446024 22941876

[19] Welz A, Reinhard I, Alpers GW, Kuehner C (2018). Happy thoughts: Mind wandering affects mood in daily life. Mindfulness.

[20] Taruffi L, Pehrs C, Skouras S, Koelsch S (2017). Effects of sad and happy music on mind-wandering and the default mode network. Sci. Reports.

[21] Woerther F (2008). Music and the education of the soul in plato and aristotle: homoeopathy and the formation of character. The Class. Q..

[22] Hsu DY, Huang L, Nordgren LF, Rucker DD, Galinsky AD (2015). The music of power: perceptual and behavioural consequences of powerful music. Soc. Psychol. Pers. Sci..

[23] Elvers, P., Fischinger, T. & Steffens, J. Music listening as self-enhancement: Effects of empowering music on momentary explicit and implicit self-esteem. *Psychol. Music*. 0305735617707354 (2017).

[24] Zentner M, Grandjean D, Scherer KR (2008). Emotions evoked by the sound of music: Characterization, classification, and measurement. Emot..

[25] Thompson ER (2007). Development and validation of an internationally reliable short-form of the positive and negative affect schedule (PANAS). J. cross-cultural psychology.

[26] Bradley, M. M. & Lang, P. J. Affective Norms for English Words (ANEW): Instruction manual and affective ratings. *Tech. Rep.*, Gainesville, Florida, University of Florida, Center for the Study of Emotion and Attention (1999).

[27] Juslin PN (2013). From everyday emotions to aesthetic emotions: towards a unified theory of musical emotions. Phys. life reviews.

[28] Zatorre RJ, Salimpoor VN (2013). From perception to pleasure: music and its neural substrates. Proc. Natl. Acad. Sci..

[29] Koelsch S (2014). Brain correlates of music-evoked emotions. Nat. Rev. Neurosci..

[30] Scherer KR (2009). The dynamic architecture of emotion: Evidence for the component process model. Cogn. & Emot..

[31] Frijda, N. H., Manstead, A. S. & Bem, S. (eds) *Emotions and beliefs: How feelings influence thoughts*. (Cambridge: Cambridge University Press, 2000).

[32] Koelsch S, Jäncke L (2015). Music and the heart. Eur. heart journal.

[33] Bhattacharya J, Lindsen JP (2016). Music for a brighter world: Brightness judgment bias by musical emotion. PloS one.

[34] Schulreich S (2014). Music-evoked incidental happiness modulates probability weighting during risky lottery choices. Front. psychology.

[35] Garrido S, Schubert E (2015). Music and people with tendencies to depression. Music. Perception: An Interdiscip. J..

[36] Garrido S, Eerola T, McFerran K (2017). Group rumination: Social interactions around music in people with depression. Front. psychology.

[37] Saarikallio S, Erkkilä J (2007). The role of music in adolescents’ mood regulation. Psychol. music.

[38] Carlson E (2015). Maladaptive and adaptive emotion regulation through music: a behavioral and neuroimaging study of males and females. Front. human neuroscience.

[39] Bénabou R, Tirole J (2002). Self-confidence and personal motivation. The Q. J. Econ..

[40] Fredrickson BL (2004). The broaden-and-build theory of positive emotions. Philos. Transactions Royal Soc. B: Biol. Sci..

[41] Kahneman, D. Thinking, fast and slow. New York: Farrar, Straus and Giroux p. 499 (2011).

[42] Peirce JW (2007). Psychopy — psychophysics software in python. J. Neurosci. Methods.

[43] Task Force of The European Society of Cardiology and The North American Society of Pacing and Electrophysiology (1996). Heart rate variability: standards of measurement, physiological interpretation and clinical use. Task Force of the European Society of Cardiology and the North American Society of Pacing and Electrophysiology. Circ..

[44] R Core Team. *R: A Language and Environment for Statistical Computing*. R Foundation for Statistical Computing, Vienna, Austria (2018).

[45] Fellows, I. *wordcloud: Word Clouds* R package version 2.6 (2018).

