# Supplementary material for: Heroic music stimulates empowering thoughts during mind-wandering
Source: Sci Rep. 2019 Jul 16;9:10317. doi: 10.1038/s41598-019-46266-w (PMC6635482; doi:10.1038/s41598-019-46266-w)
Supplement: Supplementary file 1 — S1 Text [file 41598_2019_46266_MOESM1_ESM.pdf]

Heroic music stimulates empowering thoughts during mind-wandering

Stefan Koelsch[1,*], Tobias Bashevkin[1], Joakim Kristensen[1], Jonas Tvedt[1], and Sebastian Jentschke[1]

[1]University of Bergen, Department of Biological and Medical Psychology, Postboks 7807, 5020 Bergen, Norway
[*]stefan.koelsch@uib.no

Supplementary Text S1: Additional statistical analyses with different cutoff-values for mind-wandering frequency

To assess whether more mind-wandering occurred during sad than heroic excerpts, the main text reports analyses where a response of -1 and higher (on a scale of -3 to 3) on the questionnaire item "How much were you thinking about the music vs. something else?" was coded as elicitation of "mind-wandering".

When using a cutoff value of 0 (where participants were thinking to an equal degree about the music and something else), mind-wandering occurred in 57.5% of all trials (heroic: 61.8%, sad 53.2%). Again, the difference between heroic and sad music was not significant: An ANOVA for repeated measures using the within-subject factors emotional expression (heroic vs. sad music) and tempo (slow, medium or fast) as well as the between-subjects factor presentation order (heroic first vs. sad first), revealed no significant difference in the degree of mind-wandering between pieces with different emotional expression ($p = .95$) or the different tempi ($p = .89$).

Importantly, also the results of the subsequent analyses on the effects of music on thought contents remained virtually the same, both when controlling for tempo of the music and when controlling for physiological arousal: significant differences were indicated for valence, arousal, motivation, and contructiveness (all with $p < .005$), while no significant differences were observed for any of the other items.

When using a cutoff value of 1 (where participants were more thinking about something else than about the music), mind-wandering occurred in 29.6% of all trials (heroic: 29.6%, sad 29.6%). Thus, again, no difference between heroic and sad music emerged.

Because only 30% of all trials remained with this cutoff value (with a total of 55 trials for heroic, and 55 trials for sad music, obtained from 62 participants), it was not possible to compute ANOVAs to evaluate effects of music on thought contents while controlling for tempo, or for physiological arousal. Thus, only $t-tests$ could be performed, which, however, indicated again the same general results, with significant differences in valence, arousal, motivation, and contructiveness (all with $p < .0001$), an no further differences.

Analysis for a cutoff value of -2 (meaning that participants were only partly mind-wandering) led to the inclusion of 93% of the cases, and thus to similar results as those reported for all items in Supplementary Table S2.

Note that the results analysing the words obtained in the free response format (shown in Figure 2) are independent of the cutoff values reported above, because that analysis included all trials in which a response was provided by a participant.
