# Supplementary material for: Heroic music stimulates empowering thoughts during mind-wandering
Source: Sci Rep. 2019 Jul 16;9:10317. doi: 10.1038/s41598-019-46266-w (PMC6635482; doi:10.1038/s41598-019-46266-w)
Supplement: Supplementary file 2 — S2 Text [file 41598_2019_46266_MOESM2_ESM.pdf]

Heroic music stimulates empowering thoughts during mind-wandering

Stefan Koelsch[1,*], Tobias Bashevkin[1], Joakim Kristensen[1], Jonas Tvedt[1], and Sebastian Jentschke[1]

[1]University of Bergen, Department of Biological and Medical Psychology, Postboks 7807, 5020 Bergen, Norway
[*]stefan.koelsch@uib.no

Supplementary Text S2: Pilot Study for stimulus selection

Participants: Forty-three participants (20 females, mean age = 31.35, age range 16-66), recruited by email and social media, responded to an online survey designed to identify the emotional expression of musical excerpts. The survey took approximately 20 minutes to complete. The participants were not compensated for their participation.
Stimuli: To select the appropriate stimuli for the main experiment, we collected 15 musical pieces (eight with a heroic emotional expression and seven with a sad expression) that mainly originated from film, video games and classical music. To reduce the overall duration of the survey, each stimulus was shortened to 20-seconds, because this time is well sufficient to identify the emotional expression of a musical piece.[1]
Procedure: Participants were provided with the link to the online survey and used their own computers at any time and location of their convenience. The platform used was Google-forms (https://docs.google.com/forms/), since the music stimuli could be implemented (by converting them into "YouTube"-clips) in the survey. The participants were informed that the survey was about musical impressions and they were instructed to listen to the music using headphones in a quiet environment. Before the participants were exposed to the musical excerpts, they were asked to set the audio levels to a comfortable volume, by using a provided neutral music excerpt as aid, and instructed not to adjust the loudness during music exposure.

The participants were then exposed to each of the 15 music stimuli. After each music stimulus, participants evaluated the music's emotional expression using 7-point Likert scales (Supplementary Table S4 provides an overview of these questions and their translation into English). That is, the participants were asked which emotion they thought the music was supposed to express, rather than how the music made them feel. There were 10 items assessing the emotional expression of the music. Five of these ten items were selected from the Geneva Emotional Music Scale (GEMS),[2] these items were: sadness, nostalgia, power, inspired, and peacefulness. Four additional items assessed how strongly the music expressed (i) heroism and (ii) courage, as well as the perceived (iii) arousal and (iv) pleasantness. Finally, participants of the pilot study (but not of the main experiment) rated their familiarity with the pieces on a five-point scale (from 1 = "not at all, I have not heard it before" to 5 = "very well known, I have heard it before"). The presentation order of the music excerpts was randomized.

Results: The most suitable stimuli were selected based on the difference between the scores on the items assessing expressed sadness and heroism (items #3 and #4 in Supplementary Table S4); other items were also used in case that this difference was similar for several combinations of heroic and sad stimuli. For the sad condition, the selected music was rated as pleasant ($M = 5.34$, $SEM = .16$), not arousing ($M = 1.91$, $SEM = .11$), sad ($M = 5.52$, $SEM = .15$), not heroic ($M = 2.34$, $SEM = .18$), a little nostalgic ($M = 4.84$, $SEM = .21$), not powerful ($M = 3.33$, $SEM = .19$), not inspirational ($M = 3.34$, $SEM = .20$), peaceful ($M = 5.03$, $SEM = .17$), not courageous ($M = 2.71$, $SEM = .20$), and not familiar ($M = 2.29$, $SEM = .13$). For the heroic condition, the music selected was rated as slightly pleasant ($M = 4.78$, $SEM = .13$), arousing ($M = 5.43$, $SEM = .8$), not sad ($M = 2.23$, $SEM = .14$), heroic ($M = 6.08$, $SEM = .11$), not very nostalgic ($M = 3.42$, $SEM = .17$), powerful ($M = 5.67$, $SEM = .10$), inspirational ($M = 4.99$, $SEM = .15$), not peaceful ($M = 2.50$, $SEM = .13$), courageous ($M = 5.64$, $SEM = .11$), and not very familiar ($M = 2.78$, $SEM = .14$). After stimulus selection, the excerpts were grouped into three pairs (one sad and one heroic stimulus within each pair) with the highest significant difference to the opposite emotional expression and that could best be matched in tempo.

References

1. Bigand, E., Vieillard, S., Madurell, F., Marozeau, J. & Dacquet, A. Multidimensional scaling of emotional responses to music: The effect of musical expertise and of the duration of the excerpts. *Cogn. & Emot.* **19**, 1113–1139 (2005).
2. Zentner, M., Grandjean, D. & Scherer, K. R. Emotions evoked by the sound of music: Characterization, classification, and measurement. *Emotion* **8**, 494–521 (2008).
