# Supplementary material for: Heroic music stimulates empowering thoughts during mind-wandering
Source: Sci Rep. 2019 Jul 16;9:10317. doi: 10.1038/s41598-019-46266-w (PMC6635482; doi:10.1038/s41598-019-46266-w)
Supplement: Supplementary file 3 — S1 Table [file 41598_2019_46266_MOESM3_ESM.pdf]

Heroic music stimulates empowering thoughts during mind-wandering

Stefan Koelsch[1,*], Tobias Bashevkin[1], Joakim Kristensen[1], Jonas Tvedt[1], and Sebastian Jentschke[1]

[1]University of Bergen, Department of Biological and Medical Psychology, Postboks 7807, 5020 Bergen, Norway
[*]stefan.koelsch@uib.no

Supplementary Table S1
Overview of descriptive statistics for the items assessing thought content and positive-negative affect after the presentation of heroic or sad music excerpts. The reported statistics controlled for physiological arousal, and include only trials where mind-wandering occurred (using a cutoff value of -1 on the questionnaire item "How much were you thinking about the music vs. something else?").

	heroic		sad	
Item topic	M	SD	M	SD
Items assessing thought-content				
«Valence»	1.15	.11	−.06	.12
«Arousal»	1.10	.11	−.50	.11
«Motivation»	1.13	.11	−.70	.13
«Constructiveness»	.93	.10	−.04	.103
Positive-negative affect scale, short form[1]				
Positive affect	.22	.05	−.49	.05
Negative affect	−.12	.03	.08	.03
«inspired»	.51	.08	−.38	.08
«determined»	.35	.07	−.51	.07
«active»	.24	.07	−.70	.07
«afraid»	−.22	.04	.15	.04
«alert»	.18	.07	−.34	.07

References

1. Thompson, E. R. Development and validation of an internationally reliable short-form of the positive and negative affect schedule (PANAS). *J. cross-cultural psychology* **38**, 227–242 (2007).
