# Supplementary material for: Heroic music stimulates empowering thoughts during mind-wandering
Source: Sci Rep. 2019 Jul 16;9:10317. doi: 10.1038/s41598-019-46266-w (PMC6635482; doi:10.1038/s41598-019-46266-w)
Supplement: Supplementary file 4 — S2 Table [file 41598_2019_46266_MOESM4_ESM.pdf]

Heroic music stimulates empowering thoughts during mind-wandering

Stefan Koelsch[1,*], Tobias Bashevkin[1], Joakim Kristensen[1], Jonas Tvedt[1], and Sebastian Jentschke[1]

[1]University of Bergen, Department of Biological and Medical Psychology, Postboks 7807, 5020 Bergen, Norway
[*]stefan.koelsch@uib.no

Supplementary Table S2

Overview of the results from analyses of variance for items assessing thought content and positive-negative affect for all trials (i.e., regardless of whether participants reported mind-wandering for this trial or not). Items are arranged as in Table 1 to ease comparison.

Item topic	Significant effects	F-Value	Sign.	Effect size
Items assessing thought-content				
«Valence»	heroic-sad	$F_{(1,60)} = 60.80$	$p < .0001$	$d = 2.06$
«Arousal»	heroic-sad	$F_{(1,60)} = 100.90$	$p < .0001$	$d = 2.59$
«Motivation»	heroic-sad	$F_{(1,60)} = 90.74$	$p < .0001$	$d = 2.46$
«Constructiveness»	heroic-sad	$F_{(1,60)} = 38.20$	$p < .0001$	$d = 1.60$
«Meta-awareness»	no significant effects			
«Controlled focus of attention»	no significant effects			
«Self-involvement»	no significant effects			
«Involvement of others»	no significant effects			
«Temporal orientation of thoughts»	no significant effects			
«Relevance to current life-situation»	heroic-sad x tempo x pres. order	$F_{(2,58)} = 6.99$	$p = .0019$	$d = .98$
Positive-negative affect scale, short form[1]				
«positive affect»	heroic-sad	$F_{(1,60)} = 58.79$	$p < .0001$	$d = 1.98$
«negtive affect»	heroic-sad	$F_{(1,60)} = 15.51$	$p = .0002$	$d = 1.02$
«inspired»	heroic-sad	$F_{(1,60)} = 55.18$	$p < .0001$	$d = 1.92$
«determined»	heroic-sad	$F_{(1,60)} = 50.54$	$p < .0001$	$d = 1.83$
«active»	heroic-sad	$F_{(1,60)} = 67.30$	$p < .0001$	$d = 2.12$
«afraid»	heroic-sad	$F_{(1,60)} = 18.63$	$p < .0001$	$d = 1.11$
«alert»	heroic-sad	$F_{(1,60)} = 26.43$	$p < .0001$	$d = 1.33$
«ashamed»	heroic-sad	$F_{(1,60)} = 9.70$	$p = .0028$	$d = .80$
«upset»	heroic-sad	$F_{(1,60)} = 11.82$	$p = .0011$	$d = .89$
«attentive»	heroic-sad	$F_{(1,60)} = 15.85$	$p = .0001$	$d = 1.03$
«hostile»	no significant effects			
«nervous»	no significant effects			

References

1. Thompson, E. R. Development and validation of an internationally reliable short-form of the positive and negative affect schedule (PANAS). *J. cross-cultural psychology* **38**, 227–242 (2007).
