# Supplementary material for: Heroic music stimulates empowering thoughts during mind-wandering
Source: Sci Rep. 2019 Jul 16;9:10317. doi: 10.1038/s41598-019-46266-w (PMC6635482; doi:10.1038/s41598-019-46266-w)
Supplement: Supplementary file 5 — S3 Table [file 41598_2019_46266_MOESM5_ESM.pdf]

Heroic music stimulates empowering thoughts during mind-wandering

Stefan Koelsch[1,*], Tobias Bashevkin[1], Joakim Kristensen[1], Jonas Tvedt[1], and Sebastian Jentschke[1]

[1]University of Bergen, Department of Biological and Medical Psychology, Postboks 7807, 5020 Bergen, Norway
[*]stefan.koelsch@uib.no

Supplementary Table S3
Overview of the music stimuli used in the main experiment.
Column «T» denotes the tempo pair the piece belonged to (1: slow (64 BPM), 2: medium (95 BPM) or 3: high (115 BPM); blank if the music excerpt was not chosen to be used in the main experiment), column «E« denotes the emotional expression of the piece (H: heroic, S: sad).

T	E	Composer / artist	Title	Genre
1	H	Cesc Vilà, Fran Soto Epic Soul Factory	Legendary	Neo-orchestral
1	S	Gustav Holst	Venus, the bringer of peace (from: The Planets)	Orchestral
2	H	Fearless Motivation Instrumentals	Addicted to Success (Epic Instrumental)	Neo-orchestral
2	S	Ólafur Arnalds	Film credits	String Orchestra
3	H	Spirit of America Ensemble	Heroic march	Orchestral
3	S	Ennio Morricone	Hamlet	Orchestral
