# Supplementary material for: Heroic music stimulates empowering thoughts during mind-wandering
Source: Sci Rep. 2019 Jul 16;9:10317. doi: 10.1038/s41598-019-46266-w (PMC6635482; doi:10.1038/s41598-019-46266-w)
Supplement: Supplementary file 6 — S4 Table [file 41598_2019_46266_MOESM6_ESM.pdf]

Heroic music stimulates empowering thoughts during mind-wandering

Stefan Koelsch[1,*], Tobias Bashevkin[1], Joakim Kristensen[1], Jonas Tvedt[1], and Sebastian Jentschke[1]

[1]University of Bergen, Department of Biological and Medical Psychology, Postboks 7807, 5020 Bergen, Norway
[*]stefan.koelsch@uib.no

Supplementary Table S4
Questionnaire used for stimulus selection.

	Item question	Response options
1	*Synes du musikken var ubehagelig eller behagelig?*	*1: Svært ubehagelig, 7: Svært behagelig*
	Do you think the music was unpleasant or pleasant?	1: Very unpleasant, 7: Very pleasant
2	*Synes du musikken var rolig eller energisk?*	*1: Svært rolig, 7: Svært energisk*
	Do you think that the music was calm or energetic?	1: Very calm, 7: Very energetic
3	*I hvor stor grad vil du beskrive musikken som trist?*	*1: Ikke i det hele tatt, 7: Svært mye*
	To what degree do you would you describe the music as sad?	1: Not at all, 7: Very much
4	*I hvor stor grad vil du beskrive musikken som heroisk (helteaktig)?*	*1: Ikke i det hele tatt, 7: Svært mye*
	To what degree do you would you describe the music as heroic?	1: Not at all, 7: Very much
5	*I hvor stor grad vil du beskrive musikken som nostalgisk?*	*1: Ikke i det hele tatt, 7: Svært mye*
	To what degree do you would you describe the music as nostalgic?	1: Not at all, 7: Very much
6	*I hvor stor grad vil du beskrive musikken som kraftfull?*	*1: Ikke i det hele tatt, 7: Svært mye*
	To what degree do you would you describe the music as powerful?	1: Not at all, 7: Very much
7	*I hvor stor grad vil du beskrive musikken som inspirerende?*	*1: Ikke i det hele tatt, 7: Svært mye*
	To what degree do you would you describe the music as inspiring?	1: Not at all, 7: Very much
8	*I hvor stor grad vil du beskrive musikken som fredfull?*	*1: Ikke i det hele tatt, 7: Svært mye*
	To what degree do you would you describe the music as peaceful?	1: Not at all, 7: Very much
9	*I hvor stor grad vil du beskrive musikken som modig (uttrykker mot)?*	*1: Ikke i det hele tatt, 7: Svært mye*
	To what degree do you would you describe the music as courageous?	1: Not at all, 7: Very much
10	*Hørtes musikken kjent ut?*	*1: Ikke kjent, har ikke hørt den før.*<br>*2: Vet ikke, tror ikke jeg har hørt den før.*<br>*3: Litt kjent, har kanskje hørt den før.*<br>*4: Ganske kjent, tror jeg har hørt den før.*<br>*5: Veldig kjent, har hørt den før.*
	Did the music sound familiar?	1: Not at all, I have not heard it before.<br>2: I don't know, I don't think I've heard it before.<br>3: Somewhat familiar, I've might heard it before.<br>4: Quite familiar, I think I've heard it before.<br>5: Very well known, I have heard it before.
