# Supplementary material for: Heroic music stimulates empowering thoughts during mind-wandering
Source: Sci Rep. 2019 Jul 16;9:10317. doi: 10.1038/s41598-019-46266-w (PMC6635482; doi:10.1038/s41598-019-46266-w)
Supplement: Supplementary file 7 — S5 Table [file 41598_2019_46266_MOESM7_ESM.pdf]

Heroic music stimulates empowering thoughts during mind-wandering

Stefan Koelsch[1,*], Tobias Bashevkin[1], Joakim Kristensen[1], Jonas Tvedt[1], and Sebastian Jentschke[1]

[1]University of Bergen, Department of Biological and Medical Psychology, Postboks 7807, 5020 Bergen, Norway
[*]stefan.koelsch@uib.no

Supplementary Table S5
Questionnaire used to assess thought-content after being exposed to the music excerpts.

	Item question	Response options
1	*Hvor mye tenkte du på musikken vs noe annet?*	*1: Bare på musikken, 4: På både musikken og andre ting i like stor grad, 7: Bare på noe annet*
	How much were you thinking about the music vs. something else?	1: Only on the music, 4: About both the music and other things alike, 7: Only about something else
2	*Hvor mye følte du at du hadde bevisst kontroll over dine tanker?*	*1: Svært lite kontroll, 7: svært mye kontroll*
	Did you feel like you had control over where your thoughts went?	1: Very little control, 7: Very much control
3	*Hvor bevisst var du på hvor oppmerksomheten din var fokusert?*	*1: Helt bevisst, 7: Helt ubevisst*
	How aware were you of where your attention was focused?	1: Fully aware, 7: Not aware at all
4	*Var tankene dine relatert til noe som har personlig relevans for deg (for eksempel tanker om deg selv eller et forhold du har/har hatt)?*	*1: Ikke i det hele tatt, 4: Litt, 7: Veldig mye*
	Were your thoughts related to something that is personally relevant to you (e.g thoughts about your self or a relationship you have/had)?	1: Not at all, 4: A little, 7: Very much
5	*Var tankene dine relatert til andre personer?*	*1: Ikke i det hele tatt, 4: Litt, 7: Veldig mye*
	Were your thoughts related to other people?	1: Not at all, 4: A little, 7: Very much
6	*Var det du tenkte på negativt eller positivt?*	*1: Veldig negativt, 4: Nøytralt, 7: Veldig positivt*
	Was what you were thinking about rather negative or positive?	1: Very negative, 4: Neutral; 7: Very positive
7	*Var det du tenkte på (noe som ville gjort deg) rolig (avslappet) eller aktivert (oppspilt, stresset, opphisset, energisk)?*	*1: Veldig rolig, 4: Nøytralt, 7: Veldig aktivert*
	Were the contents of your thoughts about something (that would make you) calm (relaxed) or aroused (excited, stressed, agitated, energic)?	1: Very calm, 4: Neutral, 7: Very activated
8	*Var det du tenkte på relatert til fortid nåtid eller fremtiden?*	*1: Fjern fortid, 4: Nåtid, 7: Fjern fremtid, X: Tidløst*
	Were your thought contents related to the past, present or the future?	1: Distant past, 4: Present, 7: Distant future, X: Timeless
9	*Hvor relevant var det du tenkte på til din nåværende livssituasjon?*	*1: Svært lite relevant, 7: Svært relevant*
	How relevant were your thoughts for your current life situation?	1: Very little relevance, 7: Very strong relevance
10	*Hvor konstruktive eller destruktive vil du si at tankene dine var?*	*1: Veldig destruktive, 4: Nøytral, 7: Veldig konstruktive*
	How constructive or destructive was your thoughts?	1: Very destructive, 4: Neutral, 7: Very constructive
11	*Motiverte tankene dine deg til å gjøre noe passivt (f.eks. slappe av, prokrastinere) eller noe aktivt (f.eks. utføre en oppgave, sosialisere, trene)?*	1: *Veldig passiv, 4: Nøytral, 7: Veldig aktiv*
	Did your thoughts motivate you to act rather passively (e.g relax, procrastinate) or rather actively (perform a task, socialize, exercise)	1: Very passively, 4: Neutral, 7: Very actively
